# Attitude towards prophylactic surgery and effects of genetic counselling in families with *BRCA*mutations

**DOI:** 10.1054/bjoc.1999.1086

**Published:** 2000-03-06

**Authors:** T M U Wagner, R Möslinger, G Langbauer, R Ahner, the Austrian Hereditary Breast and Ovarian Cancer Group, E Fleischmann, A Auterith, A Friedmann, T Helbich, C Zielinski, E Pittermann, M Seifert, P Oefner

**Affiliations:** Division of Senology, Division of Obstetrics, Department of Obstetrics and Gynaecology, Departments of Medical Statistics, Psychiatry and Radiology, Division of Oncology, Department of Medicine I, Ludwig Boltzmann Institute for Clinical Experimental Oncology, General Hospital, University of Vienna, Waehringer Guertel 18–20, Vienna, 1090, Austria; Department of Internal Medicine III, Hanusch Hospital, Vienna, Austria; Department of Biochemistry, University of Stanford, Palo Alto, CA, 94304, USA; see Acknowledgements

**Keywords:** *BRCA1*, *BRCA2*, genetic counselling, depression, prophylactic mastectomy, prophylactic oophorectomy

## Abstract

The intent of this study was to evaluate the effect that an awareness of being a *BRCA1* or *BRCA2* mutation carrier has on the attitude towards prophylactic surgery and on developing depression symptoms. Thirty-five families were selected on the basis of previously detected *BRCA1* or 2 mutations and 90 family members were given the appropriate questionnaires. Prophylactic mastectomy (PM) was considered by 21% of the Austrian mutation carriers (29% affected and 8% non-affected carriers). The majority of affected and non-affected carriers expected PM to impair the quality of their life. Fifty per cent would undergo prophylactic oophorectomy (53% affected and 46% non-affected carriers). The self-rating depression scale indicated that following mutation result disclosure the depression scores of carriers decreased (40 baseline vs 38 after result disclosure, *P* = 0.3), whereas, for non-carriers, scores increased (36 baseline vs 40 after result disclosure, *P* = 0.05). We conclude that information about carrier status is not associated with increased depression symptoms in mutation carriers. In non-carriers, depression scores increased slightly, probably reflecting survivor guilt. The option of having PM was associated with a negative impact on the quality of life and was declined by the majority of Austrian mutation carriers. © 2000 Cancer Research Campaign


*British Journal of Cancer* (2000) **82**(7), 1249–1253
© 2000 Cancer Research Campaign
DOI: 10.1054/ bjoc.1999.1086, available online at http://www.idealibrary.com on IDEAL®

### Attitude towards prophylactic surgery and effects of genetic counselling in families with *BRCA* mutations

**TMU Wagner[1], R Möslinger[1], G Langbauer[1], R Ahner[2], the Austrian Hereditary Breast and Ovarian Cancer Group[10], E Fleischmann[1,7], A Auterith[3], A Friedmann[4], T Helbich[5], C Zielinski[6,7], E Pittermann[8], M Seifert[1] and P Oefner[9]**

[1]Division of Senology, [2]Division of Obstetrics, Department of Obstetrics and Gynaecology, Departments of [3]Medical Statistics, [4]Psychiatry and [5]Radiology, [6]Division of Oncology, Department of Medicine I, [7]Ludwig Boltzmann Institute for Clinical Experimental Oncology, General Hospital, University of Vienna, Waehringer Guertel 18–20, 1090 Vienna, Austria; [8]Department of Internal Medicine III, Hanusch Hospital, Vienna, Austria; [9]Department of Biochemistry, University of Stanford, Palo Alto, CA 94304, USA; [10]see Acknowledgements

**Summary** The intent of this study was to evaluate the effect that an awareness of being a *BRCA1* or *BRCA2* mutation carrier has on the attitude towards prophylactic surgery and on developing depression symptoms. Thirty-five families were selected on the basis of previously detected *BRCA1* or 2 mutations and 90 family members were given the appropriate questionnaires. Prophylactic mastectomy (PM) was considered by 21% of the Austrian mutation carriers (29% affected and 8% non-affected carriers). The majority of affected and non-affected carriers expected PM to impair the quality of their life. Fifty per cent would undergo prophylactic oophorectomy (53% affected and 46% non-affected carriers). The self-rating depression scale indicated that following mutation result disclosure the depression scores of carriers decreased (40 baseline vs 38 after result disclosure, $P = 0.3$), whereas, for non-carriers, scores increased (36 baseline vs 40 after result disclosure, $P = 0.05$). We conclude that information about carrier status is not associated with increased depression symptoms in mutation carriers. In non-carriers, depression scores increased slightly, probably reflecting survivor guilt. The option of having PM was associated with a negative impact on the quality of life and was declined by the majority of Austrian mutation carriers. © 2000 Cancer Research Campaign

**Keywords**: *BRCA1*; *BRCA2*; genetic counselling; depression; prophylactic mastectomy; prophylactic oophorectomy

Mutation analysis of the *BRCA1* and *BRCA2* genes has become widely available to hereditary breast and ovarian cancer families (HBOC) or breast-cancer-only families (HBC) in North America and Europe. To date, hundreds of these families have been identified with *BRCA* mutations and the majority has received counselling regarding the test results. Follow-up care of mutation carriers has focused on cancer surveillance and surgical options (Burke et al, 1997). However, little is known about the impact carrier status has on the development of depression, or the resulting changes in the individual's body image (Lerman et al, 1996, 1997; Croyle et al, 1997; Lynch et al, 1997). In the Lerman et al (1996) study population, 18% stated their intent to undergo prophylactic mastectomy (PM) and 33% stated their intent to under prophylactic oophorectomy (PO). The motivational issues involved in deciding for or against prophylactic surgery, however, still await detailed study. Similarly, hardly anything is known about the feelings associated with this decision or their effect on the individual's quality of life in general.

The major aim of this study was to study the attitude of mutation carriers in regard to PM and PO. Furthermore, it assessed the short-term effect of knowledge about carrier status on the occurrence of depression.

*Received 10 March 1999*
*Revised 22 July 1999*
*Accepted 14 September 1999*

*Correspondence to:* T. Wagner

### PATIENTS AND METHODS

### Study population

A total of 138 individuals, from 35 families, that had been selected and counselled through the genetic counselling service of the Division of Senology, University of Vienna, were enrolled on the basis of previously detected *BRCA1* or *BRCA2* mutations. None of the family members had received the results of the BRCA testing. All individuals who requested their results were informed about this study. Family members who elected to participate gave their written consent. Prior to mutation result disclosure and counselling study participants received the first questionnaire of this study. The sequence in which the various elements of the survey were conducted is depicted in Figure 1.

All female mutation carriers are examined every 6 months for the purposes of cancer surveillance and psychosocial follow-up. The observation period for this study began in January 1996 and is currently still being continued. This study was approved by the University of Vienna Institutional Review Board.

### Attitude of mutation carriers towards the option of PM and/or PO

Female mutation carriers received a questionnaire comprising 12 items concerning PM and 11 items concerning PO to be rated on a five-point scale. Since the relevant literature does not provide a standardized questionnaire on this subject, two special questionnaires were developed on the basis of past conversations with

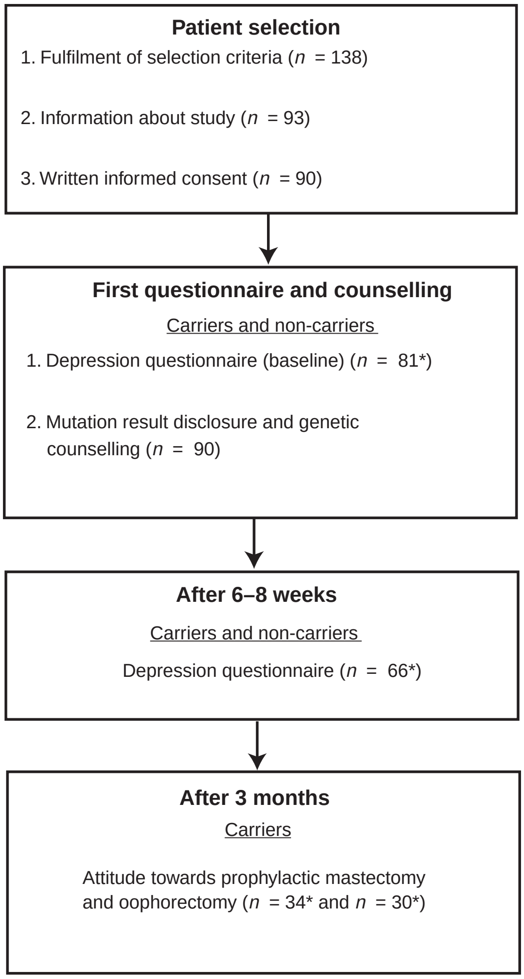

**Figure 1** Study design. *Some respondents chose to return certain questionnaires not at all or with a number of questions left unanswered mutation carriers, thereby providing a better comprehension of the attitude that Austrian mutation carriers demonstrate towards PM and PO. This questionnaire addressed the following issues: general willingness to undergo surgery, willingness to undergo PM/PO, surveillance vs PM/PO, feelings associated with PM/PO, effect on the quality of life and motivation regarding potential PM/PO.

### Depression symptoms

The validated self-rating depression scale (SDS) (Zung, 1986) consisted of 20 items requiring patients to indicate whether each one applied ‘never’, ‘occasionally’, ‘frequently’ or ‘always’. This scale has a split-half reliability of $r = 0.73$ and has been shown to correlate with the Hamilton depression scale. It is associated with clinical ratings of the severity of depression. On this measure, scores ranging from 1 to 40 are considered normal, mild depression falls between 41 and 47, mild to severe between 48 and 55, with scores above 55 indicating severe depression.

### Statistical analysis

For description of the data, frequencies and relative frequencies were given. Continuous variables were expressed as means ± standard deviation (s.d.) The ‘SDS-Index’ was computed according to Zung (1986). Wilcoxon two-sample tests were used to compare carriers versus non-carriers and affected versus non-affected carriers. The SAS® statistical software system was used for computation. A $P$-value < 0.05 was considered to indicate statistical significance.

### RESULTS

Of 138 family members from 35 mutation families counselled in regard to hereditary breast- and ovarian cancer, and subsequently tested for *BRCA1* and *BRCA2* mutations, 93 (67%) family members requested the test results. Of these, 90 (97%) family members agreed to participate in the study and received the questionnaires according to Figure 1. However, some respondents chose to return certain questionnaires not at all or with a number of questions left unanswered. Fifty-nine of the study participants were mutation carriers: two male (one affected and one non-affected) and 57 female mutation carriers. Of the female mutation carriers, 33 had already developed cancer (affected carriers) and 24 were still healthy (non-affected carriers). Thirty-one were non-carriers: three male and 28 female.

### Sociodemographic factors

The average age (s.d.) of the study participants was 38 (16). All respondents were of Austrian origin, 94% were female, 73% married. All of them had completed at least 8 years of (compulsory) school education, 43% had finished secondary school with the ‘Matura’ diploma after an additional 4 years of school. All were either employed, students or active as housewives. Due to Austria’s publicly funded health care system, all participants were covered by health insurance.

### Prophylactic mastectomy (Table 1)

Thirty-four carriers (21 affected and 13 non-affected) completed the questionnaires concerning PM. All carriers showed a general willingness to undergo surgery in case of serious illness. However, PM was considered by only 21% of mutation carriers: one (8%) of the non-affected carriers and six (29%) of affected carriers. Of the latter, one had already obtained PM of the contralateral breast, and bilateral mastectomy had been performed on two because of bilateral cancer before learning about their carrier status. After mutation result disclosure, one affected carrier had bilateral mastectomy after unilateral cancer and two intended to do so. Ninety-two per cent of non-affected carriers and 80% of the affected carriers showed a preference for surveillance. The following feelings were caused by or associated with PM: 69% of non-affected carriers experienced anxiety and 38% felt helpless, 46% regarded PM as an invasion of their privacy. The majority of affected and non-affected carriers were afraid PM would impair the quality of their life. In regard to what would favourably influence their decision to undergo PM, 62% of affected carriers named the death of close relatives due to breast cancer, 52% the reduced risk of developing cancer and 48% their fear of dying of cancer. Fifty-two per cent of all affected and 69% of non-affected carriers were first informed about the PM option during the course of the information and counselling sessions at our department (data not shown).

*© 2000 Cancer Research Campaign*

**Table 1** Attitude of female *BRCA* mutation carriers toward prophylactic mastectomy (PM), *n* = 34[a]

| **Items** | **Affected carriers, *n* = 21** | | **Non-affected carriers, *n* = 13** | |
|---|---|---|---|---|
| | **I + II + III** | **IV + V** | **I + II + III** | **IV + V** |
| General willingness to undergo surgery | 21 | 0 | 13 | 0 |
| Attitude towards PM | 6 | 15 | 1 | 12 |
| Surveillance vs PM, considering that surveillance does not provide complete protection | 17 | 4 | 12 | 1 |
| Feelings caused by/associated with PM | | | | |
| 1. Anxiety | 13 | 8 | 9 | 1 |
| 2. Helplessness | 8 | 13 | 5 | 2 |
| 3. Invasion of privacy | 11 | 10 | 6 | 3 |
| Effect of PM on the quality of life | | | | |
| 1. General | 15 | 2 | 6 | 1 |
| 2. Female identity | 16 | 4 | 6 | 2 |
| 3. Sexuality | 13 | 7 | 7 | 1 |
| Motivation in favour of PM | | | | |
| 1. Future plans | 8 | 10 | 1 | 8 |
| 2. Reduced risk of developing cancer | 11 | 8 | 4 | 4 |
| 3. Fear of dying of BC | 10 | 5 | 6 | 3 |
| Decision to undergo PM influenced by the option of reconstructive surgery | 7 | 11 | 4 | 9 |
| Reconstructive surgery will definitely be sought following potential PM | 8 | 11 | 9 | 3 |

I = certainly, II = probably, III = likely, IV = unlikely, V = does not apply. There were no significant differences between the two groups for any item (Fisher's exact test). [a]Some respondents chose to return certain questionnaires with a number of questions left unanswered.

**Table 2** Attitude of female BRCA mutation carriers toward prophylactic oophorectomy (PO), *n* = 30[a]

| **Items** | **Affected carriers, *n* = 17** | | **Non-affected carriers, *n* = 13** | |
|---|---|---|---|---|
| | **I + II + III** | **IV + V** | **I + II + III** | **IV + V** |
| General willingness to undergo surgery | 17 | 0 | 13 | 0 |
| Attitude towards PO | 9 | 7 | 6 | 7 |
| Surveillance vs. PO, considering that surveillance does not provide complete protection | 13 | 3 | 12 | 1 |
| Feelings caused by/associated with PO | | | | |
| 1. Anxiety | 8 | 6 | 7 | 2 |
| 2. Helplessness | 6 | 8 | 3 | 3 |
| 3. Invasion of privacy | 6 | 8 | 4 | 5 |
| Effect of PO on the quality of life | | | | |
| 1. General | 9 | 2 | 4 | 4 |
| 2. Female identity | 10 | 6 | 2 | 6 |
| 3. Sexuality | 9 | 7 | 2 | 6 |
| Motivation in favour of PO | | | | |
| 1. Future plans | 7 | 6 | 3 | 5 |
| 2. Reduced risk of developing cancer | 10 | 5 | 7 | 2 |
| 3. Fear of dying of BC | 10 | 5 | 6 | 3 |
| In case of PO also hysterectomy | 10 | 5 | 10 | 3 |

I = certainly, II = probably, III = likely, IV = unlikely, V = does not apply. There were no significant differences between the two groups for any item (Fisher's exact test). [a]Some respondents chose to return certain questionnaires with a number of questions left unanswered.

### Prophylactic oophorectomy (Table 2)

Thirty carriers (17 affected and 13 non-affected) completed the questionnaires concerning PO. Fifty per cent of all carriers showed a positive attitude towards PO (53% affected and 46% non-affected carriers). As in the case of PM, 76% of affected and 92% of non-affected carriers preferred surveillance. In both groups some participants showed a positive attitude towards PO, but they also marked in the following question a preference for surveillance. Subsequent to becoming part of this study, one healthy and four affected carriers had undergone PO. Fifty-three per cent of non-affected carriers experienced fear in conjunction with the idea of PO. The majority of affected and 31% of non-affected carriers showed concern about a potential impairment of the quality of their lives. The majority of affected and non-affected carriers agreed with the listed motivations in favour of PO.

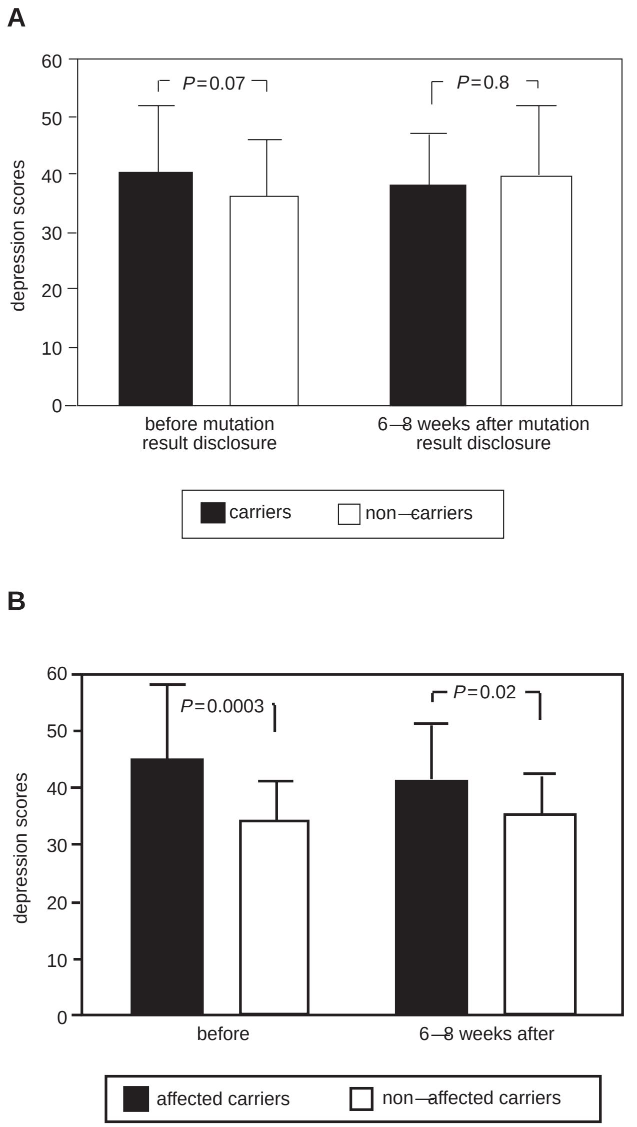

**Figure 2** (A) Depression scores of carriers versus non carriers before and 6–8 weeks after mutation result disclosure. (B) Depression scores of affected carriers versus non-affected carriers before and 6–8 weeks after mutation result disclosure

### Depression symptoms

At baseline, prior to being counselled in regard to their carrier status, SDS questionnaires from 81 participants were available. Thirty-nine per cent of all carriers (47% affected carriers and 29% non-affected carriers) and 19% of non-carriers showed mild to severe depression. There was no significant difference between carriers and non-carriers at baseline ($P$ = 0.07) (Figure 2A). However, between affected and non-affected carriers a highly significant difference could be noticed ($P$ = 0.0003) (Figure 2B).

Six to 8 weeks after mutation result disclosure, the SDS questionnaire was returned by 66 patients. Overall scores were similar between carriers and non-carriers ($P$ = 0.8). Affected carriers still presented a significantly higher depression score than non-affected carriers ($P$ = 0.02). The overall score between baseline and 6 weeks after mutation results disclosure decreased for carriers ($P$ = 0.3; Wilcoxon signed rank test), whereas depression scores for non-carriers increased ($P$ = 0.05).

### DISCUSSION

### Prophylactic surgery

At 21%, the percentage of Austrian mutation carriers who would undergo PM conforms to previously published data. Lerman et al (1996) and Lynch et al (1997) found a percentage of 18% and 35% respectively, in a North American collective, whereas in the Netherlands, Meijers-Heijboer et al (1997) determined a percentage of 57%. In our study population the prevailing rejection of PM (79%) reflects the expectation that this procedure will impair the quality of the woman's life (female identity, relationship, sexuality, leisure activity and emotional balance). In addition, PM tended to be associated with negative feelings such as fear and the invasion of one's privacy. In our findings, it was predominantly affected carriers who opted for PM but only one of the non-affected carriers. This result might be specific to the Austrian attitude towards prophylactic surgery. The assumption that non-affected Austrian carriers are disinclined to have a 'healthy' organ removed was supported by our findings. Regarding motivation for PM, only 30% of the non-affected carriers named the reduced risk of developing cancer (as opposed to 52% of the affected carriers), or the fear of dying of breast cancer (48% of the affected carriers). In this context, it is important to note that even PM does not offer 100% certainty (Hartmann et al, 1999). It remains to be seen to which degree this negative attitude towards PM was related to the fact that 59% of all carriers were first advised of the option of PM in the course of the information and counselling session. In the USA and in The Netherlands, on the other hand, patients have better access to information on this subject via the media and, consequently, tend to be more familiar with it.

The significantly higher degree of acceptance of PO (53% of affected carriers and 46% of non-affected carriers) was due to the patients' expectation that the quality of their lives was not likely to be impaired (in particular as far as female identity was concerned). On the one hand, negative feelings associated with PO tended to be less pronounced, while on the other hand motivation to undergo PO was perceived as stronger. Among the study population of Lerman et al (1996) and Lynch et al (1997) the acceptance of PO was determined to be 33% and 76%, respectively, whereas Meijers-Heijboer et al (1997) found a rate 61%. Certainly, the rate of acceptance of PO also reflects the high standard of available information about the limited diagnostic possibilities and doubtful prognosis in the face of ovarian cancer.

### Depression symptoms

Prior to disclosure of the result of mutational analysis, 39% of all carriers showed depressive symptoms. The high frequency of depressive symptoms prior to counselling is probably due to the high percentage of already affected carriers (55%) (Schover, 1991; Thompson and Shear, 1998). Six to 8 weeks after mutation result disclosure, overall scores of carriers were slightly decreased. No doubt, the reasons for this are somewhat complicated. First, learning about one's test result may reduce prolonged uncertainty

 *© 2000 Cancer Research Campaign*

and thereby enhance quality of life independent of carrier status, as proved to be the case with genetic testing for Huntington's disease (Wiggins et al, 1992; Bundey, 1997). This effect has a particular bearing on our study population because the majority of family members had already decided to undergo testing prior to genetic counselling. In this context, contributing factors may lie in the psychological robustness of those individuals choosing to take the test in the first place (Lerman et al, 1998), and the adherence to a careful protocol, as observed previously with Huntington's disease (Codori et al, 1994). Secondly, the majority of our carriers (55%) were already affected with cancer at the time of this study. For these women, learning about their carrier status did not actually represent a new development. Thirdly, one should not disregard the fact that HBOC/HBC families show a considerable awareness of the possibility of genetic predisposition. Most family members usually know several relatives who have developed or succumbed to breast or ovarian cancer and, therefore, they have come into repeated contact with this issue.

Surprisingly non-carriers showed an increase of depression scores after mutation result disclosure. In contrast, other studies (Lerman et al, 1998, Dudok deWit et al, 1998) found a decrease in depression scores in non-carriers from families with hereditary breast and ovarian cancer. However, the failure to experience relief after being identified as a non-carrier has been described for Huntington's disease. Tibben et al (1992) and Huggins et al (1992) presented data of non-carriers that express survivor guilt, emotional numbness and difficulty in coping with the effects of the test results on the family system. Our data suggest that survivor guilt might also be present in Austrian non-carriers and it will need further investigation with questionnaires addressing survivor guilt.

We conclude that in Austria, prophylactic surgery continues to be a comparatively new concept. The questionnaires specifically developed to address this subject illustrate the difference in the patients' reactions: the option of having PM performed was associated with fear and a negative impact on the quality of life by affected and non-affected individuals alike. Prophylactic oophorectomy was accepted by 50% of the participants; in general, it was not associated with an impairment of the quality of life. These results stress the importance of conducting counselling for surgical options with great care and consideration.

### ACKNOWLEDGEMENTS

This work was supported by grants from the NIH R01 HG01707, the 'Medizinischer Fond des Bürgermeisters der Stadt Wien', the 'Wiener Krebshilfe', the Ludwig-Boltzmann Institut für Klinisch-Experimentelle Onkologie, and Bundeskanzleramt, Sektion VI, Abteilung 9, Radetzkystrasse 2, 1030 Wien. We thank the families that participated in this study. Members of the Austrian Hereditary Breast and Ovarian Cancer Group are:

H Concin, Department of Obstetrics and Gynaecology, 6900 Bregenz, Austria
W Doeller, Department of Surgery, 9400 Wolfsberg, Austria
A Haid, Department of Surgery, 6800 Feldkirch, Austria
AH Lang, Department of Internal Medicine, 6800 Feldkirch, Austria
P Mayer, Department of Oncology, 5020 Salzburg, Austria
E Petru, Department of Obstetrics and Gynaecology, University of Graz, 8010 Graz, Austria
E Ropp, Specialist for Obstetrics and Gynaecology, 9020 Klagenfurt, Austria

### REFERENCES

Bundey S (1997) Few psychological consequences of presymptomatic testing for Huntington disease. *Lancet* **349:** 4

Burke W, Daly M, Garber J, Botkin J, Kahn MJE, Lynch P, McTiernan A, Offit K, Perlman J, Petersen G, Thomson E and Varricchio C (1997) Recommendations for follow-up care of individuals with an inherited predisposition to cancer. *JAMA* **277:** 997–1003

Codori AM, Hanson R and Brandt J (1994) Self-selection in predictive testing for Huntington's disease. *Am J Med Genet* **54:** 167–173

Croyle RT, Smith KR, Botkin JR, Baty B and Nash J (1997) Psychological responses to *BRCA1* mutation testing. preliminary findings. *Health Psychol* **16:** 63–72

Dudok deWit AC, Duivenvoorden HJ, Passchier J, Niermeijer MF, Tibben A and Workgroup, OMotRLG (1998) Course of distress experienced by persons at risk for an autosomal dominant inheritable disorder participating in a predictive testing program: an explorative study. *Psychosom Med* **60:** 543–549

Hartmann LC, Schaid DJ, Woods JE, Crotty TP, Myers JL, Arnold PG, Petty PM, Sellers TA, Johnson JL, McDonnell SK, Frost MH, Jenkins RB, Grant CS and Michels VV (1999) Efficacy of bilateral prophylactic mastectomy in women with a family history of breast cancer. *N Engl J Med* **340**: 77–84

Huggins M, Bloch M, Wiggins S, Adam S, Suchowersky O, Trew M, Klimek M, Greenberg C, Eleff M, Thompson L, Knight J, MacLeod P, Girard K, Theilmann J, Hedrick A and Hayden M (1992) Predictive testing for Huntington disease in Canada: adverse effects and unexpected results in those receiving a decreased risk. *Am J Med Genet* **42**: 508–515

Lerman C, Narod S, Schulman K, Hughes C, Gomez-Caminero A, George B, Gold K, Trock B, Main D, Lynch J, Fulmore C, Snyder C, Lemon SJ, Theresa C, Tonin P, Gilbert L and Lynch H (1996) *BRCA1* testing in families with hereditary breast-ovarian cancer. *JAMA* **275**: 1885–1892

Lerman C, Schwartz MD, Lin TH, Hughes C, Narod S and Lynch HT (1997) The influence of psychological distress on use of genetic testing for cancer risk. *J Consul Clin Psychol* **65**: 414–420

Lerman C, Hughes C, Lemon SJ, Main D, Synder C, Durham C, Narod S and Lynch HT (1998) What you don't know can hurt you: adverse psychologic effects in members of *BRCA1*-linked and *BRCA2*-linked families who decline genetic testing. *J Clin Oncol* **16**: 1650–1654

Lynch HT, Lemon S, Durham C, Tinley ST, Connolly C, Lynch JF, Surdam J, Orinion E, Slominski-Caster S, Watson P, Lerman C, Tonin P, Lenoir G, Serova O and Narod S (1997) A descriptive study of *BRCA1* testing and reactions to disclosure of test results. *Cancer* **79**: 2219–2228

Meijers-Heijboer EJ, van Geel AN, Seynaeve C, Logmans A, Bartels C, Tilanus-Linthorst M, Halley DJJ, van den Ouweland A, Wagner A, Majoor-Krakauer D, Niermeyer MF, Devilee P and Klijn JGM (1997) Uptake presymptomatic DNA test and preventive measures in families with inherited breast- and/or ovarian cancer. *Am J Hum Genet Suppl* **61**: A74

Schover LR (1991) The impact of breast cancer on sexuality, body image, intimate relationship. *CA-Cancer J Clin* **41**: 112–120

Thompson DS and Shear MK (1998) Psychiatric disorders and gynecological oncology: a review of the literature. *Gen Hosp Psychiat* **20**: 241–247

Tibben A, Vegter van der Vlis M, Skraastad M, Frets P, van der Kamp J, Niermeijer M, van Ommen G, Roos R, Rooijmans H, Stronks D and Verhade F (1992) DNA-testing for Huntington's disease in The Netherlands: a retrospective study on psychosocial effects. *Am J Med Genet* **44**: 94–99

Wiggins S, Whyte P, Huggins M, Adam S, Theilmann J, Bloch J, Sheps S, Schechter M and Hayden M (1992) The psychological consequences of predictive testing for Huntington's disease Canadian Collaborative Study of Predictive Testing. *New Engl J Med* **327**: 1401–5

Zung WK (1986) Zung self-rating depression scale and depression status inventory. In: *Assessment of Depression*, Sartorius N and Ban T (eds), pp 221–231. Springer: Berlin

© 2000 Cancer Research Campaign 
